# Development of a model of *Ascaris Suum* antigen-induced pulmonary inflammation in nonhuman primates

**DOI:** 10.1186/1476-9255-10-S1-P16

**Published:** 2013-08-14

**Authors:** Andrea G Bree, Franklin J Schlerman, Michael D Wadanoli, I-Ming Wang, Samuel J Goldman, Joseph P Sypek

**Affiliations:** 1 Inflammation and Remodeling Research, Pfizer Cambridge Massachusetts, 02140, USA

## 

Airway inflammation is one of the hallmarks of asthma and can contribute to airway hyperresponsiveness. Using cynomolgus monkeys (*Macaca fascicularis*) that are naturally sensitized to *Ascaris suum* antigen in the wild, we developed a reproducible model of acute airway inflammation following segmental *A.suum* antigen challenge. A pediatric bronchoscope was used to perform bronchoalveolar lavage (BAL) for the collection of BAL cells and fluid (BALF). The bronchoscope was also used for the administration of a segmental lung *A.suum* challenge. Baseline BAL was performed on one lung prior to segmental antigen challenge on the opposite lung. BAL on the challenged lung was performed 24 hours post-challenge to assess pulmonary inflammation. As compared to baseline lavage, a single airway challenge with *A.suum* antigen, at a dose of 0.50 or 0.75 mg/challenge, resulted in a reproducible pulmonary inflammation. The inflammation was characterized by an increase in total BAL cell counts and increased eosinophils. From concentrated BALF samples, taken 24 hours post *A.suum* antigen challenge, there was also a trend towards increased IL-5 and eotaxin as measured by ELISA. In some studies, animals were treated with two doses of the steroid, dexamethasone (DEX), 1 mg/kg by IM injection, prior to antigen challenge. In these studies, administration of DEX prior to antigen challenge prevented airway inflammation. These data show that segmental challenge with *A. suum* antigen produced an acute pulmonary eosinophilic inflammation that was prevented by pretreatment with the steroid, dexamethasone. This model should be useful for testing the efficacy of selected drug candidates, as compared to proven anti-inflammatory therapy, in blocking the pulmonary inflammatory response to *A. suum* in a large animal model.

